# Development of Structurally Graded Alumina–Polymer Composites as Potential Orthodontic Bracket Materials

**DOI:** 10.3390/biomimetics10040227

**Published:** 2025-04-05

**Authors:** Yin Mun Wong, Anthony J. Ireland, Bo Su

**Affiliations:** 1 Child Dental Health, Bristol Dental School, University of Bristol, 1 Trinity Walk, Avon Street, Bristol BS2 0PT, UK; tony.ireland@bristol.ac.uk; 2 Biomaterials Engineering Group, University of Bristol, Dorothy Hodgkin Building, Whitson Street, Bristol BS1 3NY, UK

**Keywords:** aesthetic orthodontic brackets, ceramic–polymer composites, gelation-freeze casting, sedimentation

## Abstract

To create an orthodontic bracket material combining the favourable properties of ceramic and polymer while minimising their limitations, graded porous ceramic scaffolds were created using unidirectional gelation-freeze casting, following which the pores were infiltrated with polymer. Two processing parameters were investigated: (1) sedimentation times of 0, 8, and 24 h, with ceramic solid loading of 20 vol.% and 2.5 wt.% gelatine concentration, and (2) ceramic solid loadings of 15, 20, and 25 vol.% with a fixed 2.5 wt.% gelatine concentration and an 8 h sedimentation time. The graded ceramic structures demonstrated porosity gradients ranging from 9.86 to 63.84 vol.%, except those with 25 vol.% ceramic solid loading at 8 h sedimentation. The Al_2_O_3_-UDMA/TEGDMA composites had compressive strengths of 60.25 to 120.92 MPa, modulus of elasticity of 19.84 to 35.29 GPa, and fracture toughness of 0.78 to 1.78 MPa·m^1/2^. The values observed were between those of dense ceramic and pure polymer. Statistical analysis was conducted using Excel^®^ 2019 (Microsoft^®^, Washington, DC, USA). Means, standard deviations, and 95% confidence intervals (CI) were calculated at a significance level of α = 0.05, alongside polynomial regression to evaluate relationships between variables. Composites with 20 vol.% ceramic solid loading at 8 h sedimentation displayed promising potential for further clinical validation.

## 1. Introduction

The global trend of growth in adult orthodontics [1,2,3,4] has heightened the significance of aesthetics in orthodontic appliances [5,6]. Beyond the immediate visual appeal, materials used in an aesthetic orthodontic bracket must also demonstrate biocompatibility, exhibit sufficient resistance to mechanical and environmental forces, and be able to maintain colour integrity. Although considerable efforts have been made to improve on the metallic look of conventional labial fixed orthodontic brackets, including the adoption of non-metallic materials such as polymers (i.e., polycarbonate and polyurethane) [7,8,9] and ceramics (i.e., alumina in mono- or poly-crystalline form), challenges persist in balancing the durability of the material [9,10,11,12] and risk of enamel damage both in use and during final bracket removal [12,13,14,15].

Polymeric brackets offer good initial aesthetics but are susceptible to unfavourable colour changes and compromised mechanical stability from the effects of water plasticisation [16,17,18]. In addition, their poor abrasion resistance [10,11,19] and creep under constant load [9], means they are unsuitable for anything other than a very short course of orthodontic treatment [20]. Conversely, ceramic brackets exhibit superior colour stability and durability and can last the more usual two-year course of orthodontic treatment. However, their renowned hardness can lead to rapid and deleterious wear of opposing tooth enamel [21]. Also, their low fracture toughness and brittle nature [22] not only increase the risk of catastrophic failure during treatment, but on completion of treatment, at the time of debonding, there is the ever-present risk of tooth enamel fracture as the brackets are removed [23].

To address these challenges, one innovative approach has been to combine the beneficial properties of both the polymer and ceramic materials, while minimising their individual drawbacks. One such design included the incorporation of a thin layer of polycarbonate (PC) mesh on the ceramic orthodontic bracket base. This design targets bond failure between the PC shim and the ceramic bracket, making the debonding process easier and reducing the risk of enamel fracture on completion of treatment [24,25]. At the same time, the oral-facing bracket comprises ceramic with its aesthetic appeal and improved longevity in clinical use. However, anecdotally, the distinct boundary between the ceramic and polymer phase frequently resulted in premature delamination.

More recent work by Al Jawoosh et al. (2020) [26] demonstrated the potential of gelation-freeze casting in developing biomimetically inspired alumina–polycarbonate (Al_2_O_3_-PC) composites, where the upper and lower borders of the resultant structures were ceramic- and polymer-rich, respectively, and with a variable ceramic–polymer gradient in between. In this way, a less distinct boundary is seen between the polymer and the ceramic. The tooth-facing side is polymer-rich to facilitate ease of debonding and the oral-facing surface is ceramic-rich for longevity during treatment. The characterisation values of the formed composites were favourable when measured against pure alumina, polycarbonate, and human enamel, offering the prospect that such a material might be suitable for use in orthodontic bracket fabrication. However, the maximum observed porosity range, and consequently the ceramic–polymer gradient across the height of the resultant composites, was limited to approximately 40%, which may not be sufficient to achieve the desired balance of functional performance required for advanced orthodontic applications [27].

The research presented here focused on manipulating the parameters of the gelation-freeze casting process to increase the ceramic–polymer gradient within the vertical direction of the composite structure. By investigating the effects of the initial ceramic solid loading and sedimentation time, the study aimed to establish a reproducible methodology for producing a structurally graded ceramic–polymer composite, with mechanical characteristics suitable for application in aesthetic orthodontic brackets.

## 2. Materials and Methods

**Figure 1:**
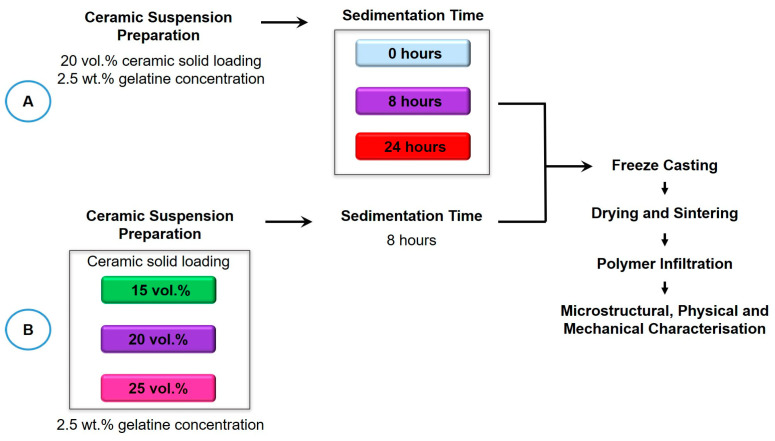
Workflow of methods. Workflow A investigated the sedimentation rate with an initial ceramic solid loading of 20 vol.% and 2.5 wt.% gelatine concentration. Workflow B investigated the effect of initial ceramic solid loading with a 2.5 wt.% gelatine concentration and a fixed 8 h sedimentation rate.

The research investigated the effects of slurry sedimentation at 0, 8 and 24 h (Figure 1 Workflow A). The initial ceramic solid loading and gelatine concentration were kept at 20 vol.% and 2.5 wt.%, respectively. Additionally, the effects of ceramic loading (15, 20, and 25 vol.%) were investigated at an optimised sedimentation time of 8 h, maintaining a gelatine concentration of 2.5 wt.% (Figure 1 Workflow B).

### 2.1. Preparation of Ceramic Slurries

Aqueous ceramic suspensions for freeze casting were prepared by mixing alumina powder (~0.5 µm; CT 3000 SG, Almatis, Frankfurt, Germany) at varying solid loadings (15, 20 and 25 vol.%) in deionised water, along with dispersant (0.6 wt.%; Dolapix CE 64, Zschimmer-Schwarz Chemie GMBH, Lahnstein, Germany). The resultant ceramic mixtures were ball-milled (SRT6, Stuart, Staffordshire, UK) at 400 rpm for at least 24 h, then roller-mixed (SLS 4234, SLS, Nottingham, UK) in a 60 °C oven (Carbolite, Derbyshire, UK) to integrate liquid gelatine (2.5 wt.%; G 1890-Type A, Sigma-Aldrich, Dorset, UK) and octanol degassing agent (0.1 wt.%; Thermo Fisher Scientific, Cheshire, UK).

After 4 h of mixing, the ceramic–gelatine slurries were sieved, poured into preheated sets of moulds (polymethyl methacrylate (PMMA) tube-fixed to a copper conduction base) to a standardised height of 20 mm, and allowed to rest in the oven at 60 °C for one of the sedimentation periods: 0, 8 and 24 h. The slurry-filled moulds were subsequently refrigerated at 7 °C for gelatinisation.

### 2.2. Unidirectional Freeze Casting

The moulds were freeze-casted at −10 °C using a pre-chilled customised setup, as described by Preiss et al. (2012) [28], maintained with a closed feedback loop integrating a temperature control device (TPC-2000, Tempco, IL, USA), a thermocouple (Type J, Watlow, MI, USA) and a band heater (MI, Watlow, MI, USA).

Frozen ceramic samples were sublimated at −55 °C under 10^−1^ mbar pressure for a minimum of 24 h/sample. The dried samples were then sintered in a temperature-controlled furnace (Model BRF17/4M, Elite Thermal Systems Ltd., Leicestershire, UK), with a sequential heating process: 400 °C at 2 °C/min for 2 h, 1530 °C at 10 °C/min for 10 min and 1550 °C at 1 °C/min for 2 h, then cooled overnight to room temperature.

### 2.3. Polymer Infiltration

For microstructural characterisation and Vicker’s hardness testing, the sintered porous Al_2_O_3_ scaffolds were infiltrated with low viscosity methylene blue-dyed epoxy using a vacuum impregnation system (Cast N’ Vac 1000, Buehler, IL, USA), at approximately 4 × 10^−1^ psi for 15 min.

A separate batch of sintered Al_2_O_3_ scaffolds (20 vol.% ceramic solid loading, 8 h of sedimentation (C20-S8)) was infiltrated with urethane dimethacrylate/triethylene glycol dimetharcylate (UDMA/TEGDMA) at 4:1 wt. ratio, to test for compressive strength, Young’s modulus, and fracture toughness. Pre-treatment involved oxygen plasma exposure (20 sccm, 100% power; Femto Plasma Surface Technology, Diener electronics GmbH & Co., Ebhausen, Germany), followed by silane coupling grafting with 3-(trimethoxysilyl)propyl methacrylate (γ-MPS) (Sigma Aldrich, Dorset, UK), to improve ceramic–polymer adhesion. Benzoyl peroxide (1 wt.%; Luperox, Sigma-Aldrich, UK) was added as a thermal activator to initiate the polymerisation process. The infiltration process utilised the vacuum impregnation system (Cast N’ Vac 1000, Buehler, IL, USA) described earlier. Post-infiltration, the Al_2_O_3_ scaffolds were heat treated at 60 °C for 1 h, 65 °C for 1 h, 70 °C left overnight, and finally at 120 °C for 2 h before being allowed to cool to room temperature, for complete polymerisation.

### 2.4. Sample Preparation for Characterisation

Polymer-infiltrated Al_2_O_3_ samples (⌀ 45 mm) were cut to appropriate dimensions (width × breadth × thickness/length) for testing using a high-speed precision cutter (Accutum-50, Struers, Birmingham, UK) with a diamond blade (15 LC IsoMet diamond wafering blade, Buehler, IL, USA), and polished manually to a 1 µm finish with a grinding machine (Tegra Pol 15, Struers, Birmingham, UK). Surface contaminants were removed by submerging the samples in deionised water within glass beakers and treating them in an ultrasonic water tank (Grant Scientific, Cambridgeshire, UK) for 30 min at 40 °C. The samples were measured using a digital calliper (Absolute Digimatic, 87 Mitutoyo, Hampshire, UK) and sectioned into three layers; (1) the ceramic-rich; closest to the conduction base, 5 mm from the conduction base, (2) the ceramic–polymer; 10 mm from the conduction base, and (3) the polymer-rich; furthest away at 15 mm from the conduction base, for the subsequent physical and mechanical characterisation in the direction of the freeze-casting.

### 2.5. Microstructural Characterisation

Two Al_2_O_3_-epoxy composite samples from each experimental group were imaged using an optical light microscope (Leica DMLB, NJ, USA) and scanning electron microscope (SEM; JSM-IT300, Jeol, Tokyo, Japan) in horizontal and sagittal cross-sections. Backscatter images were captured on the SEM, the non-conductive ceramic samples were sputter-coated with a 10 nm coating of electrically conductive gold–palladium mixture (Au/Pd) under vacuum using a sputter coater (AGB7234-DRY, Agar Scientific, Rotherham, UK). Image analysis was conducted using Fiji-Image J 2.7.0 software (Fiji, Japan) with a Modular Image Analysis (MIA) plugin developed by the Life Science Faculty of the University of Bristol (UoB).

### 2.6. Vicker’s Hardness

Three Al_2_O_3_-epoxy composite samples (10 × 10 × 2 mm) of each layer in the experimental groups were tested using a digital micro indenter (HVS-10000ZT, Jinan Hensgrand Instrument Co Ltd., Jinan, China). Each sample had six indentations made with a minimum clearance of 2 mm. The load was adjusted between HV 1.0 and HV 0.3 as required for 20 s, parallel to the direction of freeze casting. The average length of two diagonal indentations was measured using a calibrated light microscope integrated with the micro indenter, excluding any irregular indentations following the Standards of Advanced Technical Ceramics, BSEN843-4: 2005 [29].

Vickers hardness (HV) was calculated using the following formula:

(1) $$HV\approx 1.854\;(\frac{F}{D^{2}})$$

With F being the applied load measured in kilograms–force and D being the average indentation length measured in μm. Calculations were independent of the size of the indenter.

Hardness in gigapascal (GPa) was derived using the following formula:

GPa = HV × 0.009807(2)

### 2.7. Porosity and Ceramic Fraction Through Archimedes’ Principle

Three Al_2_O_3_ scaffolds (36 × 36 × 5 mm) of the ceramic-rich, ceramic–polymer and polymer-rich layers in the C20-S8 group were weighed using Archimedes’ principle of buoyancy (ASTM C373-16) [30] by converting the analytical balance ( MC1 AC 210 S, Sartorius, Göttingen, Germany) using a density kit (YDK 01, Sartorius, Göttingen, Germany).

The measurement of porosity was determined using the following equation:

Porosity (%) = (Wet weight-Dry weight)/(Wet weight-Suspended weight) × 100%(3)

and the volume fraction of ceramic was calculated by subtracting porosity:

Ceramic volume fraction (%) = 100 − Porosity(4)

### 2.8. Compressive Strength and Modulus of Elasticity

Six Al_2_O_3_-UDMA/TEGDMA composite samples (2.5 × 2.5 × 5 mm) of each layer in the C20-S8 group were tested on the Universal Testing Machine (Z020, Zwick/Roell, Ulm, Germany), with the comparatively polymer-rich surface positioned at the top. A force parallel to the freezing direction was applied at 1 mm/min until failure using a load cell with 2000 N capacity. Compressive strength (σ) was expressed as

σ = F/A(5)

where F is the failure force and A the cross-sectional area of the sample. The generated stress–strain curves were subsequently used to determine Young’s modulus (E), measured as the straight-line gradient in the elastic deformation region or calculated using the following equation:

(6) E=(F/A)/(ΔL/L)

where ΔL is the change in length of the object and L is the original length of the object.

### 2.9. Fracture Toughness

Six Al_2_O_3_-UDMA/TEGDMA composite samples (2 × 4 × 15 mm) of each C20-S8 group layer were prepared per the ASTM standard E1820-18, USA [31], with a centred notch (1.7 mm deep). With a diamond suspension-coated razor blade, the notch was extended 0.2–0.3 mm from the edge and its total length was measured using imaging software (cell Sens 1.5, Olympus, Southend-on-Sea, UK) and a research grade optical microscope (Leica DMLB, Teaneck, NJ, USA) at 40× magnification. These samples were subjected to three-point bend testing on a support span of 12.5 mm and at a testing speed of 60 μm/min until failure using the Universal Testing Machine (Z020, Zwick/Roell, Ulm, Germany).

The fracture toughness (KIC) was calculated according to the formula below:

(7) $$KIC=\left[\frac{F\times L}{B^{\frac{1}{2}}W^{\frac{2}{3}}}\right]\;f\left(\frac{a}{w}\right)$$

where F is the force, L is the support span length, a is the crack/notch length, and W and B are the width and the breadth of the Al_2_O_3_-UDMA/TEGDMA sample, respectively.

f (a/w) is the geometrical factor calculated through

(8) $$f\;\left(\frac{a}{w}\right)=\frac{3{\left(\frac{a}{w}\right)}^{\frac{1}{2}}\times \left[1.99-\left(\frac{a}{w}\right)\left(1-\frac{a}{w}\right)\left(2.15-3.93\left(\frac{a}{w}\right)+2.7{\left(\frac{a}{w}\right)}^{2}\right)\right]}{2\left(1+2\frac{a}{w}\right){\left(1-\frac{a}{w}\right)}^{\frac{3}{2}}}$$

### 2.10. Statistical Analysis

The data were analysed using Excel^®^ 2019 (Microsoft^®^, USA) and presented as means, standard deviations, and 95% confidence intervals (CI) of the mean calculated at a predetermined significance level of α = 0.05. Polynomial regression analysis was utilised via Excel’s trendline feature to model and evaluate the relationship between variables.

## 3. Results

**Figure 2:**
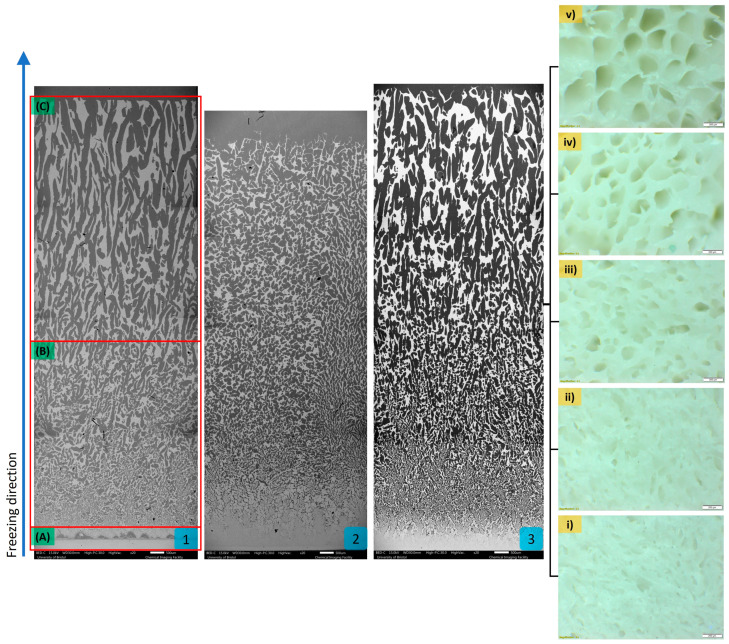
Sagittal cross-sectional view of SEM images at ×20 magnification showing the variance of freeze-cast microstructure in 20 vol.% ceramic solid loading and 2.5 wt.% gelatine concentration Al_2_O_3_-epoxy sample at three time points; (1) 0 h, (2) 24 h, and (3) 8 h. Three distinct zones were identified based on their density and pore architecture. Zone (**A**) closest to the conduction base where the freezing begun, consisted of a densely packed ceramic layer. The sample gradually became more porous in the subsequent sections, Zone (**B**) short pores with random orientation and in Zone (**C**) long pores orientated parallel to the freezing direction. Horizontal cross-section of the C20-S8 Al_2_O_3_-epoxy composite sample at ×4 magnification viewed through an optical microscope at (i) 2 mm, (ii) 4 mm, (iii) 8 mm, (iv) 12 mm, and (v) 16 mm from the conduction base demonstrated the transition of the haphazard pore architecture to honeycomb-like. The pore size increased while the ceramic wall thickness decreased along the freezing direction.

As water was used as a solvent, the formation of pores within the Al_2_O_3_ scaffolds reflected the growth of ice crystals during the freezing process. The vaporisation of the ice crystals from the ceramic scaffold during sublimation left pores that mirror their initial shape and size. Variations in the pore microstructure as depicted in Figure 2, were observed and investigated for their impact on material properties (Table 1).

### 3.1. Ceramic Scaffold Microstructure

Analysis of the horizontal cross-sections of the Al_2_O_3_ samples fabricated with 20 vol.% initial ceramic solid loading and different sedimentation times, revealed a change in pore architecture from a haphazard pattern to a hexagonal structure resembling a honeycomb, with sedimentation time affecting this transition. The change occurred higher up in the ceramic scaffold as the sedimentation time increased and became almost imperceptible at 24 h.

Distinct pore architectures were observed in composites produced with 15 and 25 vol.% ceramic solid loading at 8 h sedimentation, ranging from a combination of circular and elongated pores with dendritic-like features to randomly organised elliptical pores in the latter group.

Sagittal cross-section analysis of the ceramic structure was typical of water-based freeze-casting byproduct, with three distinct sections identified. A comparison of Al_2_O_3_-epoxy samples with and without sedimentation revealed a similar pore pattern at 8 h. However, at 24 h sedimentation, only two distinct sections were identified, albeit porous, lacking longitudinal porous channels orientated in the freezing direction. In samples with 15 vol.% ceramic solid loading, the pore architecture at 8 h sedimentation reflected the general pattern of the three distinct sections. However, with an increase in ceramic loading to 25 vol.%, the long-orientated pores typically found in the third section were not visible.

**Figure 3:**
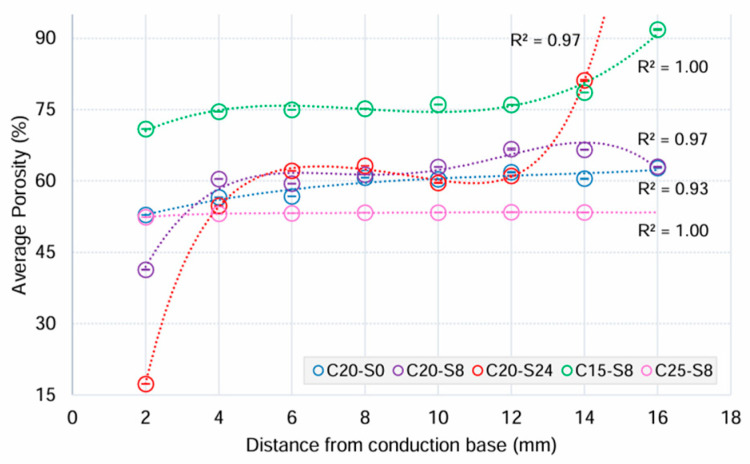
Means and 95% confidence intervals (CI) of porosity (%) from image analysis of ceramic scaffolds in each experimental group in relation to the distance from the conduction base.

Optical characterisation showed that most ceramic samples exhibited a graduated porosity profile, except in the C25-S8 group (Figure 3). With the increase in sedimentation time, a broader range of porosity was observed across the height of the samples. The porosity (vol.%) values for 20 vol.% ceramic samples ranged from 9.86 at 0 h, followed by 8 h at 25.35 and the widest range of 63.84 at 24 h. The initial ceramic solid loading also had an effect on the porosity. In the case of 15 vol.% ceramic solid loading at 8 h sedimentation, the difference in porosity between the vertical ends (20.92 vol.%) was comparable to the 20 vol.% group, but the overall porosity values (vol.%) were much higher at 70.92 (SD 0.57) to 91.84 (SD 1.51).

### 3.2. Hardness

**Figure 4:**
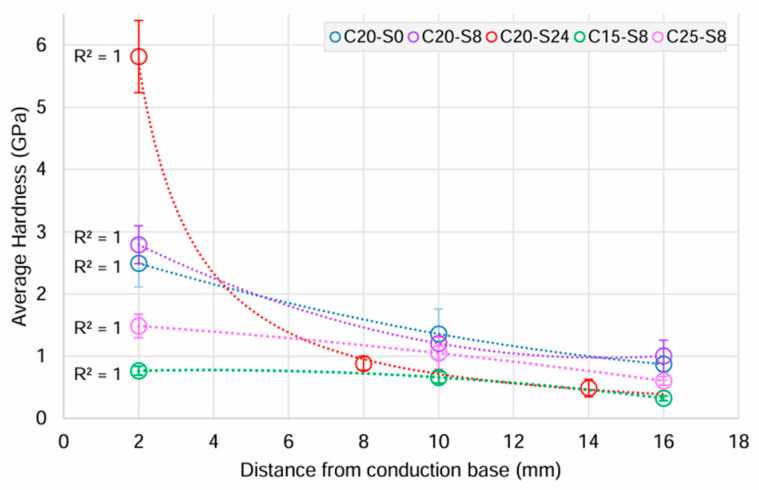
Means and 95% confidence intervals (CI) of the mean for hardness (GPa) of Al_2_O_3_-epoxy composite samples in each experimental group, in relation to the distance from the conduction base.

An examination of the Al_2_O_3_-epoxy composite hardness relative to the distance from the conduction base revealed a decline in hardness as the distance from the conduction base increased (Figure 4), potentially influenced by the ceramic–polymer content variation. The hardness value of the C20-S24 ceramic-rich layer, closest to the conduction base ((5.82 (SD1.40)) GPa) was at least twice that of the other two groups, but with the lowest value of 0.49 (SD 0.32) GPa at the polymer-rich layer. In the 0 and 8 h sedimentation groups, there was still a reduction in hardness but not as great, and from 2.49 (SD 0.82) to 0.87 (SD 0.54) and 2.79 (SD 0.7) to 1.01 (SD 0.58) GPa, respectively. A similar effect was observed with varying ceramic loading at 8 h sedimentation, with samples in the 20 vol.% initial ceramic solid loading group demonstrating the greatest resistance to plastic deformation followed by the 25 vol.% and lastly by the 15 vol.%.

### 3.3. Porosity and Ceramic Fraction Through Archimedes’ Principle

In the C20-S8 experimental group, the percentage of ceramic volume fraction decreased from the conduction base in the freezing direction. The porosity percentages were inversely proportional to the ceramic volume fractions. This indicates that as the volume of the ceramic phase decreases, there was a concomitant increase in the porosity or voids within the material.

### 3.4. Compressive Strength

Higher ceramic volume fractions in the C20-S8 experimental group Al_2_O_3_-UDMA/TEGDMA composites led to an increase in mean compressive strength from 60.25 (SD 9.16) to 120.92 (SD16.64) MPa, peaking between 50 and 52 vol.% ceramic fraction.

### 3.5. Modulus of Elasticity

Again, an increasing trend was observed in the stiffness of these C20-S8 experimental group Al_2_O_3_-UDMA/TEGDMA composite materials as the ceramic phase increased, from 19.84 (SD 4.41) to 35.29 (SD 6.7) GPa.

### 3.6. Fracture Toughness

**Figure 5:**
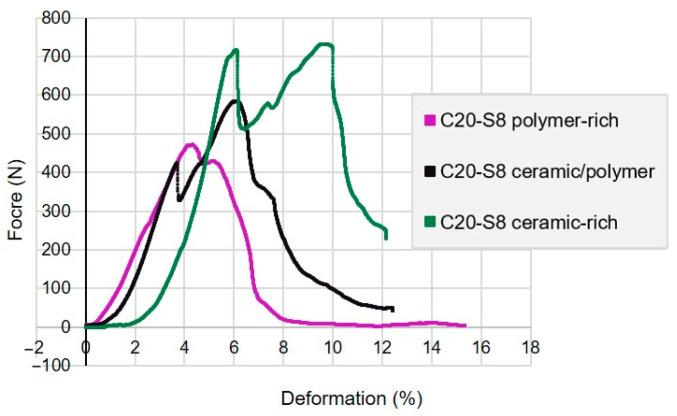
Load displacement curve of ceramic-rich, ceramic–polymer, and polymer-rich layer of Al_2_O_3_-UDMA/TEGDMA composite samples fabricated with 20 vol.% initial ceramic solid loading at 8 h of sedimentation (C20-S8) under fracture toughness testing.

As with compressive strength and modulus of elasticity, Al_2_O_3_-UDMA/TEGDMA composites of the C20-S8 experimental group displayed improved toughness with higher ceramic volume fraction, from 0.78 (SD 0.22) to 1.78 (SD 0.23) MPa·m^1/2^. The failure pattern was more gradual rather than catastrophic, indicating a more ductile behaviour of composite samples in all layers (Figure 5).

## 4. Discussion

This research investigated the effects of sedimentation time and initial ceramic loading on ceramic scaffold architecture and the mechanical properties of the fabricated ceramic–polymer composites.

Sedimentation times 0, 8, and 24 h were evaluated. Beyond 24 h, significant settling of solid particles would create a distinct boundary between the ceramic and polymer phases, rather than a graded structure.

Longer sedimentation time allowed solid ceramic particles to redistribute themselves at varying rates within the slurry, settling towards the conduction base under the influence of gravity. It is the ratio of Al_2_O_3_ particles to available water volume that will thereby influence ice crystal growth and subsequent pore formation in the ceramic scaffold [32]. This results in a greater discrepancy in water volume between the layer closest to the conduction base and the top of the suspension, thereby increasing the porosity gradient across the final freeze-cast ceramic scaffold.

The relatively higher Al_2_O_3_ concentration in the layers closest to the conduction base has two primary effects. Firstly, it leads to the creation of smaller dimension pores during freeze casting and secondly, it can inhibit particle reorganisation at the freezing front, disrupting pore formation [33]. Consequently, this results in the development of smaller, more condensed pores with a tortuous morphology and thick ceramic cell walls [26,34]. By contrast, the top layers of the suspension following sedimentation had relatively lower Al_2_O_3_ concentrations, with less hindrance to ice crystal growth, leading to larger pores with reduced ceramic wall thickness. Therefore, with increasing sedimentation time, the morphological transition from haphazard to honeycomb-like pore networks occurred further away from the conduction base. This was due to the complex nature of the altered freezing kinetics in addition to particle distribution, including the subsequent freezing rate; latent heat of diffusion; inter-particle interactions; and slurry viscosity [35,36].

The 24 h sedimentation ceramic scaffolds with a porosity range of 63.84 vol.%, favoured impregnation of the polymer phase. The range reaches a maximum of 82.7 (SD 0.6) vol.% ceramic at the lower end and 81.1 (SD 3.5) vol.% polymer at the upper end. However, these highly porous scaffolds had reduced structural integrity at the extremes of the sample [37]. The top layers all too easily chipped during handling prior to polymer infiltration, explaining why the C20-S24 Al_2_O_3_-epoxy sample (Figure 2) was shorter than the other less porous samples investigated. While an orthodontic bracket made from this material might provide a polymer-rich tooth-facing layer for safe debonding, the proportionally rich ceramic layer facing the oral environment might still pose the risk of enamel damage when in contact with opposing teeth.

With an 8 h sedimentation, reducing the ceramic solid loading from 20 to 15 vol.% increased the porosity values as there was more water available for pore formation and consequently higher porosity values were created in the final ceramic scaffold. Conversely, increasing the solid loading to 25 vol.% resulted in lower porosity. The relatively homogenous ceramic structure seen in the C25-S8 group has limited potential as a gradient structure for second-phase polymer infiltration. Although the porosity data aligned with previous findings [32], the comparison is indirect because gelatine gelation was not used, and porosity was measured as an average of the entire structure.

The hardness of the Al_2_O_3_-epoxy composite in this study was between those of pure polymer and pure ceramic, consistent with other studies [26,38]. Hardness decreased further from the conduction base as the ceramic content decreased and polymer content increased. This observation was indirectly associated with the decline in the freezing velocity during freeze casting, which affects ceramic concentration. Slower freezing front allowed time for the reorganisation of the solid particles, forming larger pores in comparison to those that were closer to the conduction base [39]. Dense pore arrangements near the conduction base usually contribute to superior hardness by providing greater resistance to deformation as a result of the increased ceramic content.

Similarly, with slurry sedimentation, the settling of dense alumina near the conduction base allowed a more compact pore structure with increased alumina density, resulting in increased hardness. This accounted for the contrast between the ceramic-rich layer ((5.82 (SD 1.40)) GPa) closest to the conduction base and the polymer-rich layer ((0.49 (SD 0.32)) GPa) furthest away at 24 h of sedimentation.

With 15 vol.% initial ceramic solid loadings, the highly porous network [37] and reduced alumina particles compromised the structural integrity of the ceramic polymer composite. In contrast, higher values of ceramic solid loading contributed to less porous structures reinforced with higher alumina concentration [32,40]. However, in the 25 vol.% solid loading group, the ceramic saturation may have resulted in some agglomeration or uneven distribution, allowing structural defects that led to inferior hardness [34,41] when compared to the 20 vol.% solid loading group.

Both ceramic–polymer composites of the C20-S0 and C20-S8 group had hardness values at the ceramic-rich end, which were comparable to enamel (2.45 to 5 GPa) [26,42,43], while maintaining reasonable hardness at the polymer-rich end. This would mean the ceramic–polymer composite could reduce the risk of enamel damage without compromising its structural integrity through a course of orthodontic treatment. The difference between 8 h of sedimentation (2.79 (SD 0.7) to 1.01 (SD 0.58) GPa) and no sedimentation (2.49 (SD 0.82) to 0.87 (SD 0.54) GPa) was subtle, possibly because the rate of sedimentation differed within the height of slurries [44].

Epoxy and UDMA/TEGDMA have similar hardness values of 0.096 GPa [38] and 0.13–0.18 GPa [45,46], respectively. This suggests that hardness values of Al_2_O_3_-UDMA/TEGDMA composites may be inferred from these Al_2_O_3_-epoxy composites. Furthermore, ceramic–polymer composites of the C20-S0 and C20-S8 group had hardness values comparable to 30 vol.% and 35 vol.% Al_2_O_3_ porous scaffolds infiltrated with UDMA/TEGDMA at 1.46 (SD 0.11) GPa and 1.62 (SD 0.09) GPa, respectively [47]. However, it is important to note that hardness value alone does not determine the overall performance of the composite material. Therefore, the most favourable ceramic sample group, C20-S8, was infiltrated with UDMA/TEGDMA and evaluated for other mechanical properties to determine its suitability for orthodontic bracket application. These samples offered a balance of porosity gradient and ease of handling.

The compressive strength of the fabricated Al_2_O_3_-UDMA/TEGDMA composites in this research ranged from 60.25 MPa at the top of the sample (45 vol.% ceramic fraction) further from the conduction base, to 120.92 MPa at the bottom of the sample (52 vol.% ceramic fraction) nearer to the conduction base. Closer to the conduction base, the interconnected network of ceramic particles is denser with fewer and smaller pores, making it more effective at distributing and transmitting compressive stresses [48,49]. However, variations in the composite composition, freezecasting process, testing methodology, and conditions can affect the observed compressive strength. Al_2_O_3_-UDMA/TEGDMA composites with 50.89 vol.% ceramic fraction [47] registered greater compressive strengths up to 253.97 MPa. Comparing the compressive strength of fabricated Al_2_O_3_-UDMA/TEGDMA composite in the present experiment with that of enamel of 62–89 MPa [50] shows them to be comparable, and able to resist permanent deformation and/or premature failure under compression.

The elastic modulus of the Al_2_O_3_-UDMA/TEGDMA composite ranged from 19.84 to 35.29 GPa, indicating a stiff material capable of maintaining its shape under load. The reinforcement effect of the ceramic–polymer scaffold in the present experiment with increasing ceramic volume fraction, as measured by Young’s modulus, was consistent with previous freeze-casting studies [47,51,52].

As with Vickers hardness, the Young’s modulus of the composite material closely matched that of natural tooth structure, falling between the values of enamel (45–105 GPa) [42,53] and dentine (17.7–21.1 GPa) [54,55]. This balance ensures that the Al_2_O_3_-UDMA/TEGDMA composite is rigid enough to provide adequate support and control during orthodontic forces, while being flexible enough to minimise the risk of damaging the tooth structures. Although significantly less rigid than relatively pure alumina at 401 GPa [51], the Al_2_O_3_-UDMA/TEGDMA composite’s rigidity range was comparable to other studies on polymer-infiltrated porous ceramic (15.53 to 30.14 GPa) [47,52,56]. 

The fracture toughness of an orthodontic bracket material is crucial for resisting crack or fracture under applied loads. The critical stress intensity factor at instability (K_IC_) quantifies fracture toughness in dental ceramics by measuring the energy concentrated at the crack tip until propagation begins. Nonetheless, the fracture behaviour of a material cannot be characterised solely by the K_IC_ test [57].

The ceramic–polymer composites in this study displayed increased toughness, with plastic deformation and a likely cellular-like fracture pattern characterised by multiple crack branches, deformation, and energy absorption during failure [58]. This fracture mode is demonstrated by materials that possess some level of toughness yet can display plastic deformation before ultimate failure. Unlike the brittle failure that occurs with pure ceramics, there was progressive failure following the second peak in each layer (Figure 5). The load drop noted after the first peak is likely due to limited crack propagation within the material [48].

The C20-S8 Al_2_O_3_-UDMA/TEGDMA composite group had mean fracture toughness values ranging from 0.78 at the top (45% ceramic volume fraction) to 1.78 MPa·m^1/2^ at the base (52% ceramic volume fraction). Within any composite, the polymer phase acts as a barrier to crack propagation, leading to crack deflection and branching. This leads to a greater energy requirement for the crack to propagate and thereby improves the fracture toughness. Increasing the polymer fraction generally increases the fracture toughness [47], but may compromise material strength, if the ceramic volume is reduced below a threshold.

Dense monocrystalline and polycrystalline alumina have fracture toughness values at 2.1–2.5 MPa·m^1/2^ [59,60] and 3.5–4.4 MPa·m^1/2^ [61,62], respectively, while enamel has a fracture toughness range of 0.67–0.95 MPa·m^1/2^ [63]. Al_2_O_3_-UDMA/TEGDMA composite in this research were between those of dense alumina and enamel, offering improved resistance to crack propagation compared to natural enamel. This is advantageous as orthodontic bracket material must withstand mastication and manipulation forces throughout orthodontic treatment, particularly at bracket tie wings, and yet not be so hard as to adversely wear the opposing teeth. The fracture toughness range of the Al_2_O_3_-UDMA/TEGDMA composite was lower than those described in other studies (2.54–4.86 MPa·m^1/2^) [46,47] with higher ceramic volume percentages (30–35 vol.% and 70–76 vol.%), and different fabrication techniques, which may partially explain the observed differences.

The study’s in vitro findings suggest that ceramic–polymer composites could greatly advance the development of aesthetic orthodontic brackets, potentially improving both clinicians’ and patients’ perceptions and acceptance of these brackets. However, further in vivo validation is needed to confirm their clinical effectiveness and safety in the oral environment.

## 5. Conclusions

Gravitational sedimentation influenced the ceramic–polymer gradient structures produced using unidirectional gelation-freeze casting. The Al_2_O_3_-UDMA/TEGDMA composites, fabricated with 20 vol.% solid loading at 8 h of sedimentation, demonstrated optimal strength and durability ceramics with the safety of polymers during debonding. This balance minimises the adverse effects typically associated with each material when used independently, such as enamel damage from ceramic hardness and polymeric creep and low abrasion resistance. Future work could investigate bonding/debonding performance and long-term durability to enhance the composite’s potential for clinical application as an orthodontic bracket material.

## Figures and Tables

**Table 1:** Means, standard deviations (SD), and 95% confidence intervals (CI) for compressive strength (MPa), modulus of elasticity (GPa), and fracture toughness (KIC) of the ceramic-rich, ceramic–polymer and polymer-rich layer of Al_2_O_3_-UDMA/TEGDMA composite samples fabricated with 20 vol.% initial ceramic solid loading at 8 h sedimentation (C20-S8).

C20-S8		Ceramic-Rich			Ceramic–Polymer			Polymer-Rich		
		Distance from Conduction Base (mm)								
		5			10			15		
		Mean	SD	95% CI	Mean	SD	95% CI	Mean	SD	95% CI
**Porosity**	**(%)**	47.74	0.93	0.61	53.34	1.23	0.80	55.19	2.50	1.63
**Ceramic Volume Fraction**	**(%)**	52.26	0.93	0.61	46.66	1.23	0.80	44.81	2.50	1.63
**Compressive Strength**	**(MPa)**	120.92	16.64	13.31	91.20	18.23	14.59	60.25	9.16	6.79
**Modulus of Elasticity**	**(GPa)**	35.29	6.70	5.36	31.40	3.06	2.45	19.84	4.41	3.27
**Fracture Toughness—K_IC_**	**(MPa·m^1/2^)**	1.78	0.23	0.20	1.54	0.21	0.17	0.78	0.22	0.18

## Data Availability

The original contributions presented in this study are included in the article. Further inquiries can be directed to the corresponding author(s).
